# Perceptual decoupling in the sustained attention to response task is unlikely

**DOI:** 10.1007/s00221-024-06885-w

**Published:** 2024-07-03

**Authors:** Aman Bedi, Paul N. Russell, William S. Helton

**Affiliations:** 1 University of Canterbury, Chirstchurch, New Zealand; 2 Department of Psychology, George Mason University, 4400 University Drive, 3F5, Fairfax, VA 22030 USA

**Keywords:** Attention, response bias, Signal detection theory, Sustained attention, Sustained attention to response task, Trigger happiness

## Abstract

Researchers dispute the cause of errors in high Go, low No Go target detection tasks, like the Sustained Attention to Response Task (SART). Some researchers propose errors in the SART are due to perceptual decoupling, where a participant is unaware of stimulus identity. This lack of external awareness causes an erroneous response. Other researchers suggest the majority of the errors in the SART are instead due to response leniency, not perceptual decoupling. Response delays may enable a participant who is initially unaware of stimulus identity, perceptually decoupled, to become aware of stimulus identity, or perceptually recoupled. If, however, the stimulus presentation time is shortened to the minimum necessary for stimulus recognition and the stimulus is disrupted with a structured mask, then there should be no time to enable perception to recouple even with a response delay. From the perceptual decoupling perspective, there should be no impact of a response delay on performance in this case. Alternatively if response bias is critical, then even in this case a response delay may impact performance. In this study, we shortened stimulus presentation time and added a structured mask. We examined whether a response delay impacted performance in the SART and tasks where the SART’s response format was reversed. We expected a response delay would only impact signal detection theory bias, c, in the SART, where response leniency is an issue. In the reverse formatted SART, since bias was not expected to be lenient, we expected no impact or minimal impact of a response delay on response bias. These predictions were verified. Response bias is more critical in understanding SART performance, than perceptual decoupling, which is rare if it occurs at all in the SART.

... we were cut off, {so} I took two guys out to try and sneak through about two miles of enemy territory where we thought another battalion might be. There was a shadow in the dark woods with a machine pistol -- hallmark of the German noncom [non-commissioned officer]. I said ‘halt’ and the pistol swung up. My shot threw him out into a patch of sunlight, and it was a GI with a tommy gun. He was the last survivor of F company, which had been cut up the night before by flame-throwing tanks.

The above is a description of a first-hand account of an American soldier accidentally shooting another American soldier during the Second World War (Trafford 1991). In combat, policing and even civilian hunting, accidental shootings can occur when there is an abrupt visual change or stimulus onset (Biggs and Pettijohn 2022; Bridges et al. 2021; Head et al. 2017; Munnik et al. 2020; Wilson et al. 2015, 2016, 2018). In these situations, the shooter has a well prepared and practiced motor response and anticipates encountering an enemy or prey. When a non-enemy or non-prey are abruptly encountered instead, the shooter has to inhibit their pre-potent motor response in order to prevent an accident. While not identical to and much less complex than these real-world high consequence situations, psychologists and neuroscientists study a similar process of inhibition by using laboratory Go-No-Go tasks with high Go rates. Go-No-Go tasks with high Go rates result in participants developing a prepared response, a key or button press response, to be instigated when a Go stimulus appears abruptly. During these Go-No-Go tasks, there occasionally are No-Go stimuli presented which require an appropriate response withhold similar to what may be occurring during some friendly fire incidents, where the expectancy is for an enemy, but instead a friend appears. The question for scientists is in this case is what mechanism causes the erroneous response.

The Sustained Attention to Response Task (SART; Robertson et al. 1997) is a widely used high Go rate Go-No-Go task. During the SART participants are presented a sequence of randomly selected digits (1–9), where each digit is presented for 250ms followed by a mask (encircled X) for 900ms. Participants are requested to make speeded responses (press a key) to the Go digits (usually1-9, except 3) and to withhold a response to the No-Go digit (typically 3). Participants in the SART are tasked with responding as quickly and accurately as they can during the task. This is a similar situation to a combatant facing potential opponents, who must respond quickly, least they themselves get attacked, but also accurately, so they do not indiscriminately shoot their allies or non-combatants (Head et al. 2017).

Researchers have suggested the routinized responding in the original SART due to the SART’s high Go format lulls the participant into treating the stimuli mindlessly or inattentively (Cheyne et al. 2009; Christoff et al. 2009; Manly et al. 1999; Robertson et al. 1997; Schooler et al. 2011; Seli 2016; Smallwood et al. 2004, 2013). These researchers argue participants occasionally withdrawal their active, presumably, conscious attention from the stimuli, and simply respond in an automated fashion. Errors in the SART from this perspective are due to perceptual decoupling. The participant does not perceive the stimuli and instead makes automated responses in the case of commission errors. If deeply enough decoupled, the participant may stop responding altogether and make errors of omission (Cheyne et al. 2009). In perceptual decoupling, the interference is not occurring merely at the oculomotor level, but at the higher level processes of central attention (Anner-Walcher et al. 2018; Cohen et al. 2022; Malpica et al. 2020). The participant may be looking at the digits but does not fully perceive them, or at the minimum does not recognize them, before making a prepotent, automated response. Many of the researchers from the perceptual decoupling perspective employ the SART to study thought content, in particular, off-task or task-unrelated thoughts, such as mind-wandering (see for example Schmidt et al. 2024).

While not disputing the existence of perceptual decoupling in other contexts, some researchers believe the role of perceptual decoupling as an explanation of performance in the SART is exaggerated or over estimated. Instead these researchers have proposed explanations that limit the role of attention in the SART. For example, researchers have proposed speed accuracy trade-offs (Dang et al. 2018), choices between fast and slow processing strategies (Peebles and Bothell 2004), impulsivity (Helton 2009) and response bias (Bedi et al. 2023) as explanations for commission errors in the SART that do not involve perceptual decoupling. SART errors in these alternative perspectives are the result of response bias or response leniency, a motor issue, not due to a lack of awareness of the stimuli per se. Indeed from these perspectives the participant often realizes they are making a commission error while they are making the error, as they are not perceptually decoupled. Instead the error is the result of a response issue, not a perception issue. Errors of omission in this perspective may occur, not only when a participant is distracted, but when the participant is trying to shift conservatively in order to mitigate perceived commission errors (Helton et al. 2010).

A line of research which may help to determine whether perceptual decoupling or alternatively response bias plays a more significant role in commission errors in the SART is incorporating response delays into the SART. Researchers have repeatedly demonstrated that a delay in responding in the SART, either due to instructions or increasing the time necessary for making a response itself, substantially improves performance (Head and Helton 2013; Manly et al. 2000; Seli et al. 2012; Wilson et al. 2018). From a perceptual decoupling perspective delaying the response should only improve performance if the participant has time to recouple their attention. The stimuli in the original SART were presented for 250ms. This prolonged presentation time may provide enough time for participants to recouple their perception, if they were initially perceptually decoupled at stimulus onset.

Bedi and colleagues (2024) reduced the stimuli presentation time from 250ms to 50ms to discover if the response delay effect could be explained by recoupling. There was still a notable decline in commission errors with a response delay. If the participant was perceptually decoupled during initial stimulus presentation, there was no additional time to recouple their perception. The stimuli were only present for 50ms, which is about the limit at which numerical digits are reliably recognized (Dehaene et al. 1999). In this case, there was no time to realistically recover attention if the participant was initially perceptually decoupled. Instead of perceptual decoupling, Bedi and colleagues (2024) suggested alternatively there may be a dynamic alteration in response bias during the SART. The participant upon encountering the high Go nature of the task becomes extremely response lenient or “trigger happy.” Furthermore, Bedi and colleagues suggested this response leniency may be assessed by using the Signal Detection Theory (see Green and Swets 1966) metric of response criterion or bias, c. In Signal Detection Theory, c is a measure of the participant’s willingness to make a response or not, and c is separated from the metric d′, which is a measure of the participant’s ability to separate out signals (targets) from noise. Bedi and colleagues demonstrated that c does become more conservative with a response delay.

Reducing stimulus duration to the minimum necessary for stimulus identification prevents the re-engagement of attention should participants be perceptually decoupled during the 250ms stimulus duration typically employed in the SART. Participants may, however, be able to recover from perceptual decoupling if the opportunity for recovery existed after stimulus offset and before the delayed response cue. Recovery may be possible even with 50ms digit exposures if participants have access to the display during the delay interval in some temporary visual memory system such as iconic or visual sensory memory (Keogh and Pearson 2011; Sperling 1960). In order to prevent continued access to the digit display after it ceases to be visible on the screen and therefore to prevent any advantage that may accrue from recovery from perceptual decoupling, it is necessary to immediately follow the display by an effective mask (Enns and Di Lollo 2000; Kouider and Dehaene 2007). Bedi and colleagues in their study used the standard encircled X mask used traditionally in the SART.

In the present experiment the encircled X mask commonly used in the SART was replaced by a structured pattern mask (see Fig. 1) which incorporates features shown to restrict post-display processing. This mask is comprised of line segments that are designed to replicate the contour properties of the digits used as stimuli. This is done so that when the mask is overlaid on any of the 9 digits the stimulus is rendered invisible. The experiment involved a between groups design with no delay and 450ms delay groups in which 50 ms digit displays were followed by the structured pattern mask.

**Fig. 1:**
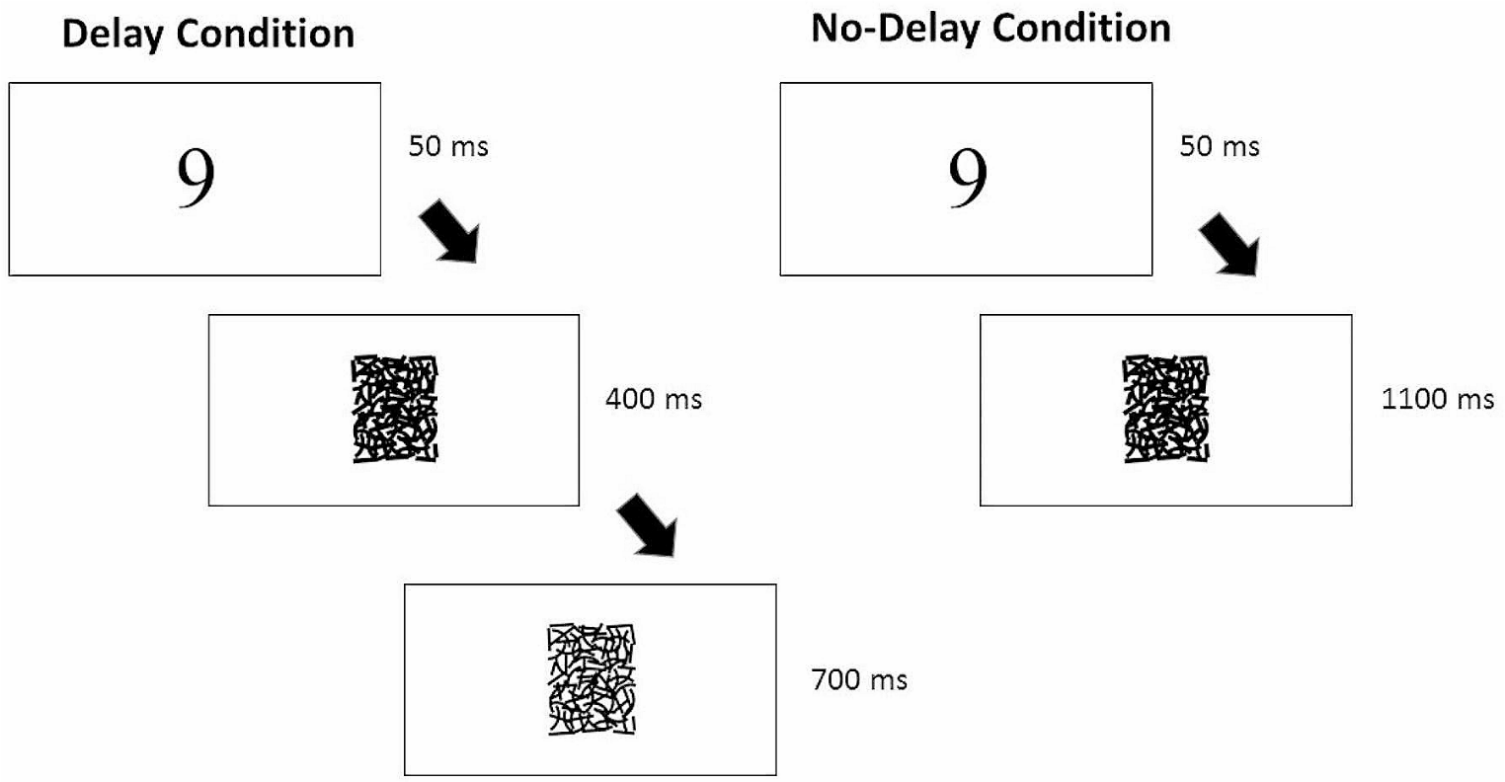
Experiment with structured mask with delay and no-delay conditions

We also incorporated two additional conditions were the response format of the SART was reversed, one with a 450ms delay and one with no delay. In these conditions the participants were instructed to respond only to the digit 3, and not to make a response to the other digit stimuli (1–9, besides 3). These reverse formatted conditions enabled us to inspect the level of participants’ perceptual coupling to the task stimuli without the possible confounding of the high Go nature of the task leading to excessive response leniency.

We expected in the SART response format a substantial impact of delay on Signal Detection Theory metric c, with a more conservative shift, in line with the previous results of Bedi and colleagues (2024). However, in the reverse SART format we expected little to no effect of a response delay on c. The reverse SART with its low Go format should not result in excessive response leniency which can be ameliorated by a response delay. In regards to d′, we expect in line with the results of Bedi and colleagues (2024), no significant impact of a response delay for either response format. The most substantial result will be for c to shift towards conservative with a response delay in the SART format.

## Method

### Participants

Seventy-eight (50 female, 28 male) participated in this experiment either for partial fulfilment of course requirements or in exchange for a NZ $20 shopping voucher. Their ages ranged from 18 to 51 years (M = 21.9 years). All participants had normal or corrected-to-normal vision. The research was approved by the University of Canterbury Human Ethics Committee.

### Apparatus

Participants were tested in groups of 3–4 seated at individual cubicles in a larger 35-cubicle psychology laboratory at the university. Each participant was seated approximately 50 cm in front of an LCD computer screen (377 mm x 303 mm, 1680 × 1050 pixels, 60 Hz refresh rate) that was mounted at eye level. Participants’ head movements were not restrained. Stimulus presentation and response accuracy and timing were achieved using E- prime 2.0 software. Responses were made using the left mouse button of a mouse connected to a serial port of an i7 PC computer running Windows 7. Mobile devices were deactivated for the duration of the experiment.

### Stimuli

Stimuli were the digits 1–9 presented in the centre of the screen in black Symbol font at 120 pixel size on a white background. The digits were each immediately followed by a thick patterned mask (see Fig. 1). The stimulus set comprised of 25 replications of the 9 digits (225 total stimuli). The stimulus presented on each trial was selected at random from the entire 225 stimulus set without replacement. In the two SART conditions (450ms delay and no delay) the digit 3 was the designated No-Go and the remaining digits were Go digits. In the two reverse SART conditions (450ms delay and no delay) the digit 3 was designated the Go and the remaining digits were No-Go. For the two 450ms delay groups the thick pattern mask changed to the thin pattern mask (see Fig. 1) 450ms after digit onset.

### Procedure

Participants were given an information sheet explaining the task and a consent form to sign upon arrival. Participants were assigned randomly during each group session to a standard SART or reverse format SART and to either a delay or no-delay condition; so one of four possible conditions. This resulted in 20 participants in the SART no delay condition, 21 participants in the SART delay condition, 20 participants in the reverse SART no delay condition, and 17 participants to the reverse SART delay condition. We were aiming for ~ 20 participants per condition in line with previous studies using similar tasks (see Bedi et al. 2024). A between participants design was employed to eliminate possible carryover effects across conditions. Participants in the delay conditions were instructed to refrain from making a response on Go trials until the lines forming the mask thinned, which occurred 450ms after digit onset (or 400ms after digit offset). Once the structured mask thinned, they were to respond as quickly as they could, but to refrain from responding to the No Go stimuli. In the no delay conditions, the participants were instructed to respond to Go digits as quickly as they could, but to refrain from responding to the No Go stimuli (no delay from stimulus onset). All digits were presented for 50ms (3 refresh cycles). After the digit was presented the thick structured mask was displayed. In the no delay conditions the thick mask appeared for 1100ms, until the next digit appeared. In the delay conditions, after the digit offset, the thick structured mask was displayed for 400ms and then was changed to the thin structured mask (which indicated they should respond) for 700ms. The digit onset to onset interval for all conditions was 1150ms. Participants in all groups completed 45 practice trials (5 presentations of each digit in a random order) and accuracy feedback was displayed visually during these practice trials. Once practice was completed and participants understood the task they were going to perform, the main task proceeded with no interruptions.

## Results

Only participants who had fewer than 25% omissions were included in the analyses. One participant was excluded from the final analysis from the SART delay condition and another participant in the reverse SART delay condition was also excluded. Two-tailed tests are reported unless noted otherwise.

### Method check: does the delay actually delay response times?

**Fig. 2:**
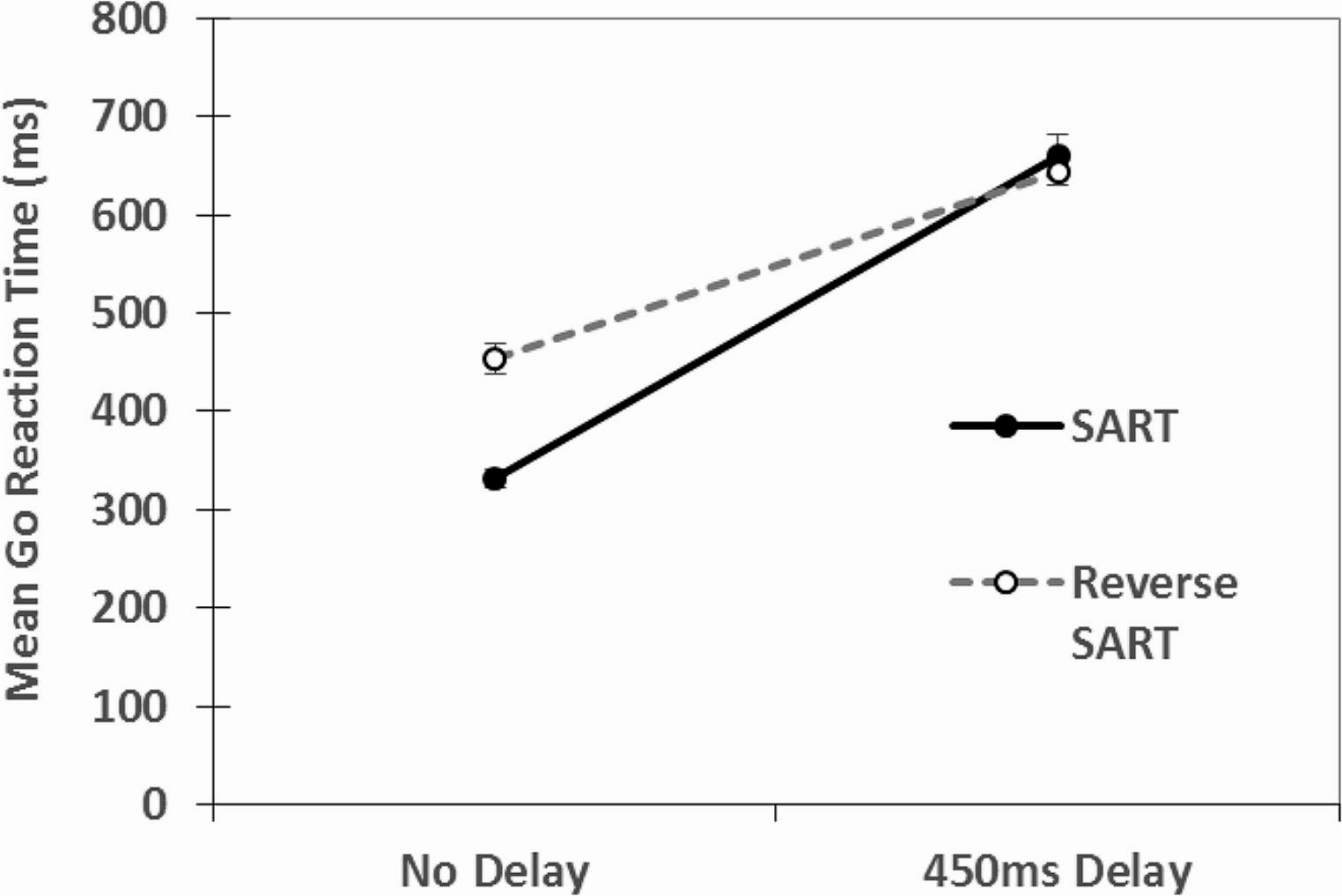
Mean Go reaction times (ms) for both the SART and Reverse SART formats for both no delay and 450ms delay conditions. Error bars are standard errors of the mean

We compared the 50ms structured mask presentation for both the SART response format and reverse-SART response format for both delay and no-delay conditions using a 2 (response format: SART vs. reverse-SART) by 2 (delay vs. no delay) ANOVA for Go reaction times. All reaction times were measured from stimulus (digit) onset. There was a significant main effect for delay, F(1,72) = 263.17, *p* < .001, ω^2^ = 0.72, there was a significant main effect for response format, F(1,72) = 10.73, *p* = .002, ω^2^ = 0.03, and there was a significant delay by response format interaction, F(1,72) = 19.37, *p* < .001, ω^2^ = 0.05. The Go reaction times (ms) for all conditions are displayed in Fig. 2. Thus, the delay manipulation led to slower RTs, as expected. The significant interaction is due to the reverse-SART format with no delay having slower RTs than the SART format. No-Go reaction times were not analysed similarly because in the reverse-SART format participants made few commission errors and thus many participants had no No-Go reaction times.

### Signal detection theory

**Fig. 3:**
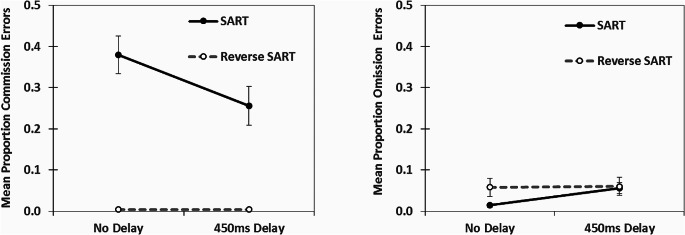
Mean proportion commission and omission errors for all four tasks. Error bars are the standard error of the mean. Where the error bars are not noticeable, the error bar is smaller than the figure marker

**Fig. 4:**
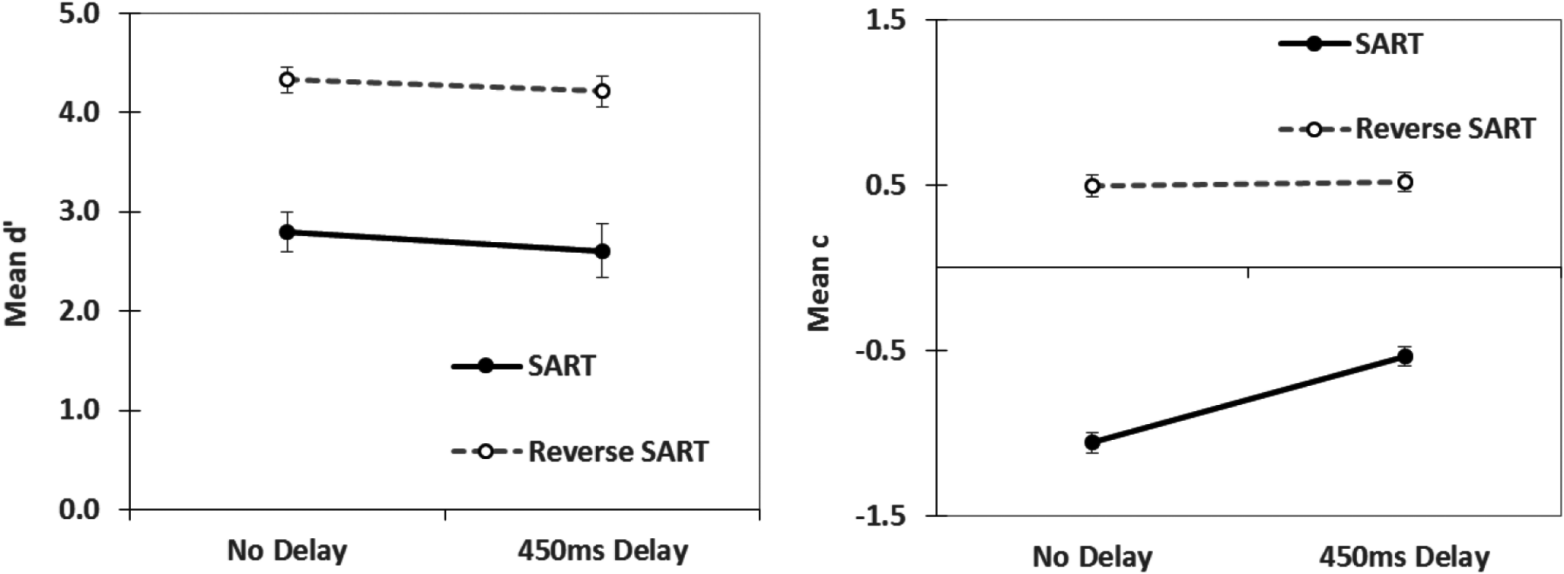
Mean d′ and c for all four tasks. Errors bars are standard errors of the mean

We calculated Signal Detection Theory metrics of d′ and c. d′ is a metric of sensitivity, the ability to separate out signals (targets) from noise, and c is a metric of response bias, the willingness to make a response or not. For the calculations of d′ and c correct responses to the Go stimuli were considered correct detections, failures to respond to Go stimuli (omissions) were considered misses, erroneous responses (commissions) to No–Go stimuli were considered false alarms and correct withholds to No–Go stimuli were considered correct rejections (see Bedi et al. 2023, 2024). The commission and omission error rates (proportions) for all conditions are displayed in Fig. 3. The calculations for d′ and c were taken from formulas provided by Stanislaw and Todorov (1999). The log linear correction was utilized for all participants to address the issue of extreme values (Hautus 1995). We then analysed differences using 2 (response format: SART vs. reverse-SART) by 2 (delay vs. no delay) ANOVAs for both d′ and c. For d′, there was a significant main effect for response format, F(1,72) = 60.34, *p* < .001, ω^2^ = = 0.44, but there was no significant main effect for delay, F(1,72) = 0.58, *p* = .45, ω^2^ = 0.00, nor a significant interaction, F(1,72) = 0.03, *p* = .86, ω^2^ = 0.00. For c, there was a significant main effect for delay, F(1,72) = 19.53, *p* < .001, ω^2^ = 0.03, a significant main effect for response format, F(1,72) = 446.37, *p* < .001, ω^2^ = 0.80, and a significant delay by response format interaction, F(1,72) = 16.13, *p* < .001, ω^2^ = 0.03. The d′ and c for all conditions are displayed in Fig. 4. Of direct interest was the comparison of d′ and c for the SART format for the no delay and the delay conditions. We, therefore, also calculated the associated Bayes Factors (Keysers et al. 2020) as evidence in support of either a null hypothesis (BF_01_) or the alternative hypothesis (BF_10_) for these comparisons. In the case of d′, the comparison for the delay versus no delay for the SART resulted in a BF_01_ = 2.86. In the case of c, the comparison of the delay versus no delay SART resulted in BF_10_ = 40295.49. When we examined the reverse SART format for the no delay and delay condition. In the case of d′, the comparison for the delay versus no delay for the reverse SART resulted in a BF_01_ = 2.70 and in the case of c, the comparison resulted in BF_01_ = 3.01. As expected, the delay only shifted response criterion c significantly conservatively in the SART format, not the reverse SART format. There was no significant impact of a response delay on d′ regardless of format.

## Discussion

In our study, we were examined the impact of a response delay with a structured mask on a modified SART with a short (50ms) stimulus presentation. This was done to examine whether the SART is subject to perceptual decoupling or instead, shifts in response bias (willingness to respond or not). Participants in this study when instructed to delay responding, did actually comply with this instruction and delayed their responses. Response times to the Go stimuli for both delay conditions in the present study were notably longer than the no delay conditions (see Fig. 2).

In regards to Signal Detection Theory metric d′, delaying a response had an insignificant impact as we predicted and was demonstrated previously in Bedi et al. (2024). Response format (SART versus reverse SART), however, did have a significant impact on d′. The Signal Detection Theory metric d′ was substantially higher in the reverse SART format than the SART format (see Fig. 4). This was due to the greater number of errors of commission in the SART format, with 38% errors of commission in the no delay SART and 26% errors of commission in the delayed SART. In the reverse SART condition both the no delay and delayed version had less than 1% errors of commission (almost 0%). Participants in the reverse SART (low Go, high No-Go) essentially made no errors of commission. Regardless, the average d′ values, 2.70, even in the SART format are high for a supposed vigilance or sustained attention task. Indeed in a meta-analysis of d′, See and colleagues (1995) found laboratory vigilance or sustained attention tasks had a d′ average of 2.43, with a 95% CI between 2.20 and 2.66. When the response format is reversed in the SART in this study, so low Go, high No Go, the average d′ is greater than 4. One interpretation of these findings is the SART stimuli (clearly presented digits) even with a shortened presentation time are simply too easy to discriminate to be considered a good candidate for a vigilance or sustained attention task.

The Signal Detection Theory metric c, had a significant interaction between delay and response format. As predicted levels of c did not differ based on a delay for the reverse SART format, but c did become clearly more conservative with a delay for the standard SART format (see Fig. 4). The SART format had an overall more liberal response bias than the reverse formatted SART and a delay only impacted response bias in the SART condition. The low Go, high No-Go format of the reverse SART format in this study was more similar to the response format of standard vigilance or sustained attention tasks (see See et al. 1995), where targets to be responded to are relatively rare, and overall response bias is conservative. In vigilance or sustained attention tasks, unlike in the SART and similar high-Go tasks, a No-Go stimulus is the most likely stimulus to occur and there is no need to inhibit a perpotent response. In the low Go, high No Go tasks, the default or likely response is no-response at all. This is also apparent in the slower Go response times in the no-delay reverse SART than the Go response times in the non-delay standard SART. The slower response times in the reverse SART no-delay condition may reflect a more conservative response bias. In the SART, the most likely stimulus to occur is a Go stimulus. The resulting lenient response bias in the SART and similar high Go tasks is substantially different from vigilance and sustained attention tasks, with their low Go nature and conservative response bias (Dang et al. 2018, 2023; Helton 2009). In the present case, we find corroborating support for Bedi et al. (2024) where response delays in the SART primarily impact response bias, c. Even with a structured mask, a response delay in the SART appears to impact c not d′.

Another finding in the present study is the relative number of errors of commission in the SART formatted tasks in comparison to the errors of omission in the reverse SART formatted tasks. In the SART the errors of commission were failures to withhold to the digit 3, whereas in the reverse SART the errors of omission were failures to respond to the digit 3. The digit 3, in both response formats, was perceptually identical. However, in the SART without a delay an error of commission was 6.6 times more likely to be made than an error of omission in the reverse SART without a delay. In the case of a response delay, this difference reduced to 4.3 times more likely. In the present study, all stimuli were only presented for 50ms and followed by a structured mask. Presumably perceptual decoupling, either due to internal or external distraction, could explain the errors of omission in the reverse SART conditions; these errors did occur about 6% of the time a 3 was presented. Advocates of the perceptual decoupling theory in the SART, however, would need to explain why the digit 3 errors are so much higher in the SART than the reverse SART format, as they are perceptually identical.

Perhaps, the repetitive responding of the SART lulls the participant into a distracted, perceptually decoupled state. Why, however, would not responding much at all, as in the reverse SART, really result in less decoupling? If anything in the high Go format the participant needs to either time their responses to onsets or be perceptually coupled enough to respond to a stimulus onset, otherwise the participant would make too many omission errors. In a low Go format (reverse SART), if anything the task is more forgiving to perceptual decoupling, as there is less requirement to continually engage with the task. The odds of needing to respond at all are lower in the low Go format. A more likely explanation is perceptual decoupling is overall rare in the SART (see also a similar point made by Dillard et al. 2014). The SART is a short, less than 5 min, perceptually easy (removing response inhibition, d′ > 4) task. Perceptual decoupling may occur in the SART, but not as frequently as errors in the SART would suggest. The errors are mostly due to something other than perceptual decoupling; we would suggest response leniency or trigger happiness (Head et al. 2017; Munnik et al. 2020; Wilson et al. 2015, 2016, 2018). Researchers should extend the duration of the SART (see for example Carter et al. 2013) in order to examine possible temporal dynamics of response criterion in the SART over time and include different stimuli, other than clearly presented numbers, that are harder to discriminate (see for example Nuechterlein et al. 1983).

## Data Availability

The dataset for the current study is not publicly available due the fact that they constitute an excerpt of research in progress but are available from the corresponding author on reasonable request.

## References

[CR1] Annerer-Walcher S, Körner C, Benedek M (2018). Eye behavior does not adapt to expected visual distraction during internally directed cognition. PLoS ONE.

[CR2] Bedi A, Russell PN, Helton WS (2023). Go-stimuli probability influences response bias in the sustained attention to response task: a signal detection theory perspective. Psychol Res.

[CR3] Bedi A, Russell PN, Helton WS (2024). Perceptual decoupling or trigger happiness: the effect of response delays and shorter presentation times on a go-no-go task with a high go prevalence. Exp Brain Res.

[CR4] Biggs AT, Pettijohn KA (2022). The role of inhibitory control in shoot/don’t-shoot decisions. Q J Exp Psychol.

[CR5] Bridges KE, Corballis PM, Spray M, Bagrie J (2021). Testing failure-to-identify hunting incidents using an immersive simulation: is it viable?. Appl Ergon.

[CR6] Carter L, Russell PN, Helton WS (2013). Target predictability, sustained attention, and response inhibition. Brain Cognition.

[CR7] Cheyne JA, Solman GJ, Carriere JS, Smilek D (2009). Anatomy of an error: a bidirectional state model of task engagement/disengagement and attention-related errors. Cogn.

[CR8] Christoff K, Gordon AM, Smallwood J, Smith R, Schooler JW (2009). Experience sampling during fMRI reveals default network and executive system contributions to mind wandering. Proc Natl Acad Sci.

[CR9] Cohen D, Nakai T, Nishimoto S (2022). Brain networks are decoupled from external stimuli during internal cognition. NeuroImage.

[CR10] Dang JS, Figueroa IJ, Helton WS (2018). You are measuring the decision to be fast, not inattention: the sustained attention to Response Task does not measure sustained attention. Exp Brain Res.

[CR11] Dang JA, Shaw TH, McKnight PE, Helton WS (2023). A closer look at warning cues on the sustained attention to response task performance. Hum Factors.

[CR12] Dehaene S, Spelke E, Pinel P, Stanescu R, Tsivkin S (1999). Sources of mathematical thinking: behavioral and brain-imaging evidence. Science.

[CR13] Dillard MB, Warm JS, Funke GJ, Funke ME, Finomore VS, Matthews G, Shaw TH, Parasuraman R (2014). The sustained attention to response task (SART) does not promote mindlessness during vigilance performance. Hum Factors.

[CR14] Enns J, Di Lollo V (2000). What’s new in visual masking?. Trends Cogn Sci.

[CR15] Green DM, Swets JA (1966). Signal detection theory and psychophysics.

[CR16] Hautus MJ (1995). Corrections for extreme proportions and their biasing effects on estimated values of d′. Behav Res Methods Instruments Computers.

[CR17] Head J, Helton WS (2013). Perceptual decoupling or motor decoupling?. Conscious Cogn.

[CR18] Head J, Tenan MS, Tweedell AJ, LaFiandra ME, Morelli F, Wilson KM, Helton WS (2017). Prior mental fatigue impairs marksmanship decision performance. Front Physiol.

[CR19] Helton WS (2009). Impulsive responding and the sustained attention to response task. J Clin Exp Neuropsychol.

[CR20] Helton WS, Weil L, Middlemiss A, Sawers A (2010). Global interference and spatial uncertainty in the sustained attention to Response Task (SART). Conscious Cogn.

[CR21] Keogh R, Pearson J (2011) Mental imagery and visual working memory. PLoS ONE:6(12) 10.1371/journal.pone.0029221 PMC3237605 22195024

[CR22] Keysers C, Gazzola V, Wagenmakers EJ (2020). Using Bayes factor hypothesis testing in neuroscience to establish evidence of absence. Nat Neurosci.

[CR23] Kouider S, Dehaene S (2007). Levels of processing during non-conscious perception: a critical review of visual masking. Philos Trans R Soc Lond B Biol Sci.

[CR24] Malpica S, Serrano A, Gutierrez D, Masia B (2020). Auditory stimuli degrade visual performance in virtual reality. Sci Rep.

[CR26] Manly T, Robertson IH, Galloway M, Hawkins K (1999). The absent mind: further investigations of sustained attention to response. Neuropsychologia.

[CR25] Manly T, Davison B, Heutink J, Galloway M, Robertson IH (2000). Not enough time or not enough attention? Speed, error and self-maintained control in the sustained attention to response test (SART). Clin Neuropsychological Assess.

[CR27] Munnik A, Näswall K, Woodward G, Helton WS (2020). The quick and the dead: a paradigm for studying friendly fire. Appl Ergon.

[CR28] Nuechterlein KH, Parasuraman R, Jiang Q (1983). Visual sustained attention: image degradation produces rapid sensitivity decrement over time. Science.

[CR29] Peebles D, Bothell D (2004) Modelling performance in the Sustained Attention to Response Task. Proc ICCM 231 236. Carnegie Mellon University/University of Pittsburgh, Pittsburgh, PA

[CR30] Robertson IH, Manly T, Andrade J, Baddeley BT, Yiend J (1997). Oops!’: performance correlates of everyday attentional failures in traumatic brain injured and normal subjects. Neuropsychologia.

[CR33] Schmidt LM, Chaieb L, Derner M, Reber TP, Fell J (2024). Side effects of monaural beat stimulation during sustained mental work on mind wandering and performance measures. Front Psychol.

[CR32] Schooler JW, Smallwood J, Christoff K, Handy TC, Reichle ED, Sayette MA (2011). Meta-awareness, perceptual decoupling and the wandering mind. Trends Cogn Sci.

[CR34] See JE, Howe SR, Warm JS (1995). Meta-analysis of the sensitivity decrement in vigilance. Psychol Bull.

[CR35] Seli P (2016). The attention-lapse and motor decoupling accounts of SART performance are not mutually exclusive. Conscious Cogn.

[CR36] Seli P, Cheyne JA, Smilek D (2012). Attention failures versus misplaced diligence: separating attention lapses from speed-accuracy trade-offs. Conscious Cogn.

[CR37] Smallwood J (2013). Penetrating the fog of the decoupled mind: the effects of visual salience in the sustained attention to response task. Can J Exp Psychol/Revue canadienne de psychologie expérimentale.

[CR38] Smallwood J, Davies JB, Heim D, Finnigan F, Sudberry M, O’Connor R, Obonsawin M (2004). Subjective experience and the attentional lapse: Task engagement and disengagement during sustained attention. Conscious Cogn.

[CR39] Sperling G (1960). The information available in brief visual presentations. Psychol Monographs: Gen Appl.

[CR40] Stanislaw H, Todorov N (1999). Calculation of signal detection theory measures. Behav Res Methods Instruments Computers.

[CR41] Trafford A (1991) Fallout from ‘friendly fire’: forgiveness after an unavoidable accident. Washington Post https://www.washingtonpost.com/archive/lifestyle/wellness/1991/02/12/fallout-from-friendly-fire/ec76bdad-cfa3-468b-8f90-494cc38b61fe/

[CR42] Wilson KM, Head J, De Joux NR, Finkbeiner KM, Helton WS (2015). Friendly fire and the sustained attention to response task. Hum Factors.

[CR44] Wilson KM, Finkbeiner KM, de Joux NR, Russell PN, Helton WS (2016). Go-stimuli proportion influences response strategy in a sustained attention to response task. Exp Brain Res.

[CR43] Wilson M, Joux NR, Finkbeiner KM, Russell PN, Retzler JR, Helton WS (2018). Prolonging the response movement inhibits the feed-forward motor program in the sustained attention to response task. Acta Psychol.

